# Drug response testing for elective carotid artery stenting: Prevalence of non-response to aspirin and clopidogrel and influence on post-interventional occurrence of cerebral ischemia

**DOI:** 10.1177/19714009251372360

**Published:** 2025-08-29

**Authors:** Farzaneh Yousefi, Dimah Hasan, Frederic De Beukelaer, Hani Ridwan, Omid Nikoubashman, Martin Wiesmann, Charlotte S. Weyland

**Affiliations:** Department of Neuroradiology, University Hospital RWTH Aachen, Aachen, Germany

**Keywords:** Carotid artery stenting, clopidogrel resistance, aspirin resistance, acute ischemic stroke

## Abstract

**Background:**

The patient-associated prevalence of Clopidogrel (CPG)—and Aspirin (ASS)—nonresponse is not well understood and varies depending on the patient population. The influence of responder status for platelet inhibition in patients eligible for carotid artery stenting (CAS) on post-interventional cerebral ischemia is unknown.

**Methods:**

We conducted a retrospective, mono-center analysis of all patients with response-test undergoing elective CAS between 2010 and 2024 and available MRI before and after CAS. Study groups were formed according to ASS- and CPG-response. Cerebral ischemia patterns were compared between study groups in univariate analysis and patient-associated co-morbidities were tested for association with drug resistance or infarction frequency.

**Results:**

In total, 50/68 (73.5%) of patients showed adequate response to ASS and CPG. Non-response to CPG was higher than to ASS (clopidogrel resistance rate: 14.8%, aspirin resistance rate: 9.2%). All patients with non-response were bridged with GP IIb/IIIa antagonist tirofiban during CAS. Under these conditions, the responder status did not influence post-interventional cerebral infarction patterns.

**Conclusion:**

Antiplatelet non-response, especially for CPG, is very frequent in patients undergoing CAS. When bridging patients with tirofiban during intervention, responder status had no influence on post-interventional cerebral infarction patterns.

## Introduction

Carotid artery stenting (CAS) has become a well-established treatment for high-grade or symptomatic carotid stenosis. CAS is a procedure with a risk of thromboembolic events, which can be approximately less than 1% (stroke and transient ischemic attacks) and up to 40% (usually as a small, non-symptomatic diffusion restriction on MRI), although it has been reduced in recent years due to the different protection methods.^1^ For prevention of these thromboembolic events and stent thrombosis, a pretreatment with Dual Antiplatelet Therapy (DAPT) should be performed, whereas data on the timing and dosage of DAPT for carotid stenting are limited.^2^ Despite the importance of DAPT, a significant number of patients exhibit resistance to commonly used medications such as clopidogrel and aspirin (ASS). This resistance can hinder the achievement of adequate platelet inhibition, underscoring the need for monitoring antiplatelet function and adjusting the medication in case of drug resistance.^3,4^

The Multiplate test is an effective method for evaluating the functional effect of aspirin and clopidogrel separately. This test determines whether the required level of platelet inhibition has been achieved to prevent thromboembolic events.^5^

In cases of drug resistance, various strategies can be used to achieve a therapeutic level of platelet inhibition. For example, administering a double dose of clopidogrel may be effective in some cases of clopidogrel resistance.^6^ Another approach is the use of tirofiban, a Glycoprotein IIb/IIIa receptor inhibitor, which is recommended as a weight-adjusted bridging therapy for clopidogrel in resistant patients undergoing neurointerventional therapies.^7^

Post-interventional infarctions are not always symptomatic. Silent infarctions, which are small, non-symptomatic Diffusion-weighted Imaging (DWI) lesions discovered incidentally on post-interventional MRI, have been observed.^8^ While the importance of these silent infarctions is not well understood, it has been shown that they increase the risk of recurring cerebrovascular events.^9^ Some studies suggest that these silent—i.e., non-symptomatic—MRI lesions could lead to cognitive impairment.^10–13^

This study aims to evaluate the rate of CPG- and ASS-resistance in patients eligible for elective CAS. Secondly, it aims to compare infarction frequency and size between patients with CPG- or ASS-resistance and patients without drug resistance.

## Methods

This monocentric retrospective study evaluated patients who underwent elective carotid artery stenting (CAS) in the cervical segment due to stenosis between 2010 and 2024. The study was approved by the local Ethics committee (local registration number (EK055-21 and EK24-223) and performed in accordance to the Declaration of Helsinki 2004. The study included patients without further treatments other than CAS, with an applicable and reliable Multiplate Analyzer® test before stent-implantation and a magnetic resonance imaging (MRI) examination performed within 9 days after stent implantation. Patient-associated comorbidities were tested for association with response status or cerebral infarction frequency.

### Patient population; inclusion and exclusion criteria

**Figure 1.**
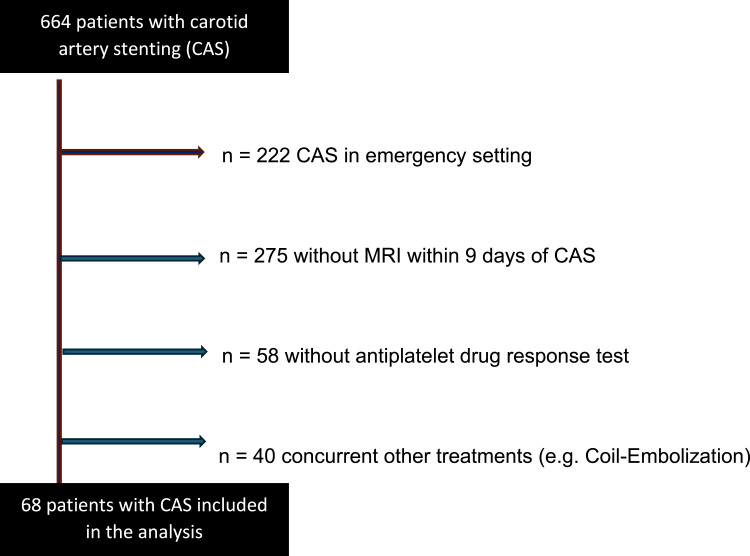
Flow chart for patient selection. Flow chart explaining the patient selection for the study analysis with exclusion criteria and number of cases excluded; CAS—carotid artery stenting.

Between 2010 and 2024, 664 patients underwent CAS. Patients without MRI in 9 days after CAS and patients without Multiplate Test immediately before CAS were excluded. Patients with tumor-related stenosis, with other concurrent treatments and with dissection as underlying pathology were also excluded—see Figure 1.

### Magnetic resonance imaging (MRI)

MRI was performed using a Siemens 1.5 Tesla or 3 Tesla scanner. The imaging protocol always included diffusion-weighted imaging and an Apparent Diffusion Coefficient (ADC) map as well as a T2 Fluid Attenuated Inversion Recovery (FLAIR) with 3 mm slice thickness.

### Platelet function testing

Assessment of clopidogrel resistance or aspirin resistance for all patients was performed using multiplate electrode impedance aggregometry (Roche Diagnostic, Mannheim, Germany). Three parameters were evaluated after stimulation with arachidonic acid (ASPI test), adenosine diphosphate (ADP test), and thrombin receptor activator peptide (TRAP test). The platelet function testing was performed immediately before the CAS procedure in the angiography suite. The necessary equipment is available in the angiography suite at all time. The test itself is performed by the neurointerventional staff. This allows for determining whether the patients might need additional therapy, such as tirofiban, immediately prior to the procedure. Based on the Multiplate test results, patients were classified as responders if they demonstrated sufficient platelet inhibition after dual antiplatelet therapy (DAPT). Responders received no further medication before or during stent implantation. Nonresponders, defined by inadequate platelet inhibition in the ASPI test or ADP test, or patients without platelet inhibition beforehand received supplemental medication with tirofiban (weight-adjusted) and/or aspirin IV (500 mg). After the intervention, all patients were prescribed aspirin and clopidogrel; in cases of clopidogrel resistance, prasugrel or ticagrelor was used as a replacement and tirofiban was stopped 6 hours after drug loading dose.

### Carotid artery stenting—local standard treatment protocol

The procedures were performed under either local anesthesia or general anesthesia using a right or left femoral access. Carotid Wallstent (Boston Scientific, Marlborough, Massachusetts, USA) was used for the majority of cases. For other cases, the following stents were implanted in order of descending frequency—Casper Stent (MicroVention, Aliso Viejo, California, USA), Enterprise stent (Codman Neuro, Massachusetts, USA), Pharos stent (Codman Neuro, Raynham, MA), Dynamic (Biotronik GmbH & Co. KG., Germany), C-Guard stent (InspireMD Inc. Boston, USA), Acculink (Abbot Inc., USA), and Acclino-Flex stent (Acandis GmbH, Pforzheim, Germany). Pre-dilatation was performed only if the stent delivery system could not pass through the stenosis. Post-dilatation was performed in case of relevant residual stenosis after stent implantation.

### Infarction classification

Infarctions or diffusion restrictions were classified into subgroups based on size (small ≤5 mm, large >5 mm) and location (cortical, subcortical, deep gray matter, and brainstem). Infarctions in the territory of non-catheterized arteries were also evaluated.

### Data analysis

The statistical analysis was performed using IBM SPSS Statistics version 26. Normal distribution for descriptive analysis was tested using the Shapiro–Wilk and Kolmogorov–Smirnov test. Quantitative variables were expressed as mean ± standard deviation (SD) or median and interquartile ranges (IQR). Categorical variables were analyzed with Chi-square or Fischer’s exact test, continuous variables were analyzed using the independent sample *t* test or Mann–Whitney U-test and correlation between two variables were analyzed using Pearson test or Spearman’s rho test, according to distribution. The risk factors such as hypertension, current smoking, former smoking, obesity, dyslipidemia, diabetes mellitus, peripheral arterial disease (PAD), alcohol abuse, and hyperuricemia were analyzed using Mann–Whitney U-test. A two-sided *p*-value of <0.05 was considered statistically significant.

## Results

Sixty-eight patients were analyzed in this study (47 male patients, 69.1%, and average age of 68.6 years).

Based on the Multiplate test results, 50 patients were categorized as responders (73.5%), 15 patients as non-responders (22.1%), and three patients who did not receive any medication before intervention were categorized as without premedication.

Of the 68 patients, 65 patients received aspirin, of which 59 patients were sensitive to aspirin and six patients had aspirin resistance (aspirin resistance rate: 9.2%). 61 patients received clopidogrel, of which 52 patients were sensitive to clopidogrel and nine patients had clopidogrel resistance (clopidogrel resistance rate: 14.75%)—see Table 1.

**Table 1.** Platelet inhibition response status of patients undergoing elective carotid artery stenting.

Resistance status	ASS	Clopidogrel
Sufficient, *n*	59	52
Not sufficient, *n* (%)	6 (9.2%)	9 (14.8%)
Without premedication, *n*	3	7

Response status was tested with Multiplate test immediately before intervention.

Patient-associated comorbidities such as arterial hypertension, current smoking, former smoking, obesity, dyslipidemia, diabetes mellitus, peripheral arterial disease (PAD), alcohol abuse, and hyperuricemia were evaluated for association with response status—see Table 2.

**Table 2.** Patient co-morbidities and their frequency according to platelet inhibition response status.

Variables	Responder	Non-responder	*p*-value^a^
Arterial hypertension, *n*	44	13	0.787
Obesity, *n*	10	1	0.464
Current smoking, *n*	14	7	0.149
Former smoking, *n*	6	3	0.448
Dyslipidemia, *n*	22	6	0.735
Hyperuricemia, *n*	4	0	0.485
Diabetes mellitus type 1 or 2, *n*	16	7	0.461
Peripheral artery disease, *n*	4	1	0.317
Alcohol abuse, *n*	2	1	0.112

^a^chi-square test (*p* values in bold = <0.05).

Risk factors such as hypertension, obesity, dyslipidemia, diabetes mellitus, peripheral arterial disease (PAD), hyperuricemia, alcohol abuse, and current or former smoking do not influence the responsiveness to CPG and/or ASS.

The analysis revealed that risk factors such as hypertension, obesity, dyslipidemia, diabetes mellitus, peripheral arterial disease (PAD), alcohol abuse, and hyperuricemia did not influence the rate of any type of infarction. However, current smoking was associated with a lower total number of infarctions (*p* = 0.026), large infarctions (*p* = 0.008), and cortical infarctions (*p* = 0.038) compared to patients without smoking—see Table 3.

**Table 3.** Association of risk factors with infarction detection after elective carotid artery stenting.

	Hypertension, asymp. Sig. (2-tailed)	Adipositas, asymp. Sig. (2-tailed)	Current smoking, asymp. Sig. (2-tailed)	Current smoking, asymp. Sig. (2-tailed)	Dyslipidemia, asymp. Sig. (2-tailed)	Hyperuricemia, asymp. Sig. (2-tailed)	Diabetes mellitus, asymp. Sig. (2-tailed)	Peripheral arterial disease, asymp. Sig. (2-tailed)	Alcohol, asymp. Sig. (2-tailed)
Total infarction	0.343	0.522	**0.026**	0.930	0.716	0.863	0.7884	0.416	0.272
Small infarction	0.69	0.426	0.165	0.645	0.859	0.708	0.590	0.658	0.330
Large infarction	0.150	0.650	**0.008**	0.264	0.362	0.783	0.869	0.454	0.867
Territorial infarction	1.000	1.000	1.000	1.000	1.000	1.000	1.000	1.000	1.000
Subcortical infarction	0.549	0.396	0.62	0.992	0.420	0.846	0.591	0.647	0.676
Cortical infarction	0.259	0.447	**0.038**	0.937	0.870	0.780	0.824	0.310	0.305
Deep gray matter	0.828	0.895	0.643	0.632	0.306	0.396	0.103	0.916	0.582
Brainstem infarction	1.000	1.000	1.000	1.000	1.000	1.000	1.000	1.000	1.000

(*p* values in bold = <0.05).

There was no difference in the number of infarctions detected by MRI between patients who received tirofiban, either because they did not respond to aspirin or CPG or they did not receive any premedication, and responders—see Table 4. The number of infarcts was not associated with the patient’s age. Balloon assisted angioplasty before stent implantation tended to increase the number of small infarcts (*p* = 0.055) and cortical lesions (*p* = 0.078). Balloon-assisted angioplasty after stent-implantation had no influence on the number of infarctions. The use of aspiration at the guide catheter as a protective method during carotid artery stent deployment and during balloon-deflation is associated with a lower rate of large infarctions in this study cohort (*p* = 0.007). In contrast, the use of a balloon guide catheter or filter wire did not result in a reduction of infarction rates in this study—please see Tables 5 and 6 in supplementary material.

**Table 4.** Detailed number of infarctions in responder and non-responder groups.

	Responder (*n* = 50)	Non-responder (*n* = 18)	Asymp. Sig. (2-Tailed)
Total infarction; *n*, median (interquartile range)	260, 2.50 (8)	109, 1.00 (6)	0.225
Small infarction; *n*, median (interquartile range)	183, 2.00 (4.25)	67, 0.50 (2.5)	0.137
Large infarction; *n*, median (interquartile range)	79, 0.00 (1.25)	42, 0.00 (2.25)	0.678
Territorial infarction; *n*, median (interquartile range)	0, 0.00 (0)	0, 0.00 (0)	1.000
Subcortical infarction; *n*, median (interquartile range)	59, 0.00 (2)	29, 0.00 (0.5)	0.258
Cortical infarction; *n*, median (interquartile range)	179, 2.00 (4.25)	82, 1.00 (3)	0.247
Deep gray matter; *n*, median (interquartile range)	8, 0.00 (0)	2, 0.00 (0)	0.587
Brainstem infarction; *n*, median (interquartile range)	0, 0.00 (0)	0, 0.00 (0)	1.000

Mann–Whitney U Test.

## Discussion

This study shows that a significant rate of patients eligible for elective CAS are drug resistant to aspirin or clopidogrel (22.1%). In our study, clopidogrel resistance was 14.8% and aspirin resistance was 9%. These results are in line with Hidayat et al., who showed that 15.8% of Indonesian ischemic stroke patients receiving clopidogrel have resistance.^14^ Yoshimura et al., shows also the rate of clopidogrel resistance of 14% and aspirin resistance of 16.3% in Asian patients with carotis stenting.^15^ Other studies demonstrated that the rate of clopidogrel might be as high as 63–66% in some populations.^16,17^ Increasing the dose of aspirin or clopidogrel may lead to overcoming resistance in some patients.^18^ Fifi et al. there was a nonsignificant decrease in thromboembolic complications in patients whose clopidogrel dosage was tailored to the assay.^6^ In addition to doubling the dose of an antiplatelet agent to overcome drug resistance, intra-venous administration of tirofiban during CAS is also an effective option.^7^ Our findings indicate that tirofiban bridging therapy successfully equalizes infarction rates between drug-resistant and drug-sensitive patients.

In this study, risk factors such as hypertension, former smoking, current smoking, obesity, dyslipidemia, diabetes mellitus, peripheral arterial disease, alcohol abuse, and hyperuricemia have no association with aspirin or clopidogrel resistance. They also have no association with any kind of cerebral infarction pattern except for current smoking. Hidayat et al. demonstrated no significant association between smoking and clopidogrel resistance, nor between diabetes mellitus and clopidogrel resistance.^14^ Similarly, Kang et al. found no link between smoking and clopidogrel resistance.^19^ In contrast, Maruyama et al. observed that ischemic stroke patients who are current smokers have a lower likelihood of developing clopidogrel resistance compared to non-smokers. They also reported no association between diabetes mellitus and clopidogrel resistance, or between hypertension and clopidogrel resistance.^20^

Patients who currently smoked had fewer total infarctions, large infarctions (more than 5 mm), and cortical infarctions compared to patients who did not smoke. In a meta-analysis by Feng et al., smoking decreased the risk of symptomatic or silent cerebral ischemia.^21^ Dig et al. demonstrated that current smoking at the time of carotid artery stenting resulted in a reduction of serious adverse events.^22^ In our study, only current smoking was associated with a lower infarction rate. There was no correlation between MRI lesions and being a former smoker. Some studies show that active cigarette smoking may enhance the antiplatelet effects of clopidogrel through the induction of the enzyme cytochrome P450 2C19 responsible for converting clopidogrel to its active form.^23,24^ In this study cohort, balloon assisted angioplasty before stent implantation tended to increase the number of small infarctions (*p* = 0.055) and cortical lesions (*p* = 0.078) while Balloon-assisted angioplasty after stent-implantation had no influence on the number of infarctions. An effect of balloon-angioplasty before stent-implantation might be provable with a bigger study cohort size. In accordance with our findings, Gray et al. demonstrated that pre-dilatation is associated with a higher incidence of stroke—15.4%—compared to a significantly lower rate of 4.3% observed in patients who did not undergo pre-dilatation without an embolic protection device within the first 30 days.^25^ Theiss et al have shown the same results that pre-dilatation led to higher periprocedural stroke rate of 4.1% versus 3.0%.^26^ Pre-dilatation probably is a surrogate marker of lesion severity but it is unclear from current data.^27^ Besli et al. demonstrated that, despite the lack of a significant increase in the incidence of major adverse events at 30 days, post-dilatation emerged as an independent predictor of silent cerebral ischemia.^28^ Nevertheless, some other studies have demonstrated that carotid artery stenting (CAS) performed without post-dilatation is both feasible and safe, and is associated with a low incidence of adverse periprocedural outcomes. However, these studies did not assess the presence of silent ischemia utilizing diffusion-weighted imaging (DWI).^29,30^ Our study shows that TAH drug resistance is a common phenomenon in patients eligible for CAS and that technical details of the intervention might play a role for ischemic events.

## Limitations

This study is limited by its single-center retrospective study design and its relatively small sample size. Population based differences in liver enzyme activity and responder status could lead to differing drug response rates in different populations. Data regarding the timing and precise dosage of the initial administration of aspirin and clopidogrel were unavailable for some patients. As the study site is testing for drug response before carotid artery stenting, non-responders are treated with tirofiban to prevent adverse events. This obscures the direct effect of antiplatelet non-response on cerebral ischemias after CAS.

## Conclusion

Drug non-response to antiplatelet medication, especially for clopidogrel, is very frequent in patients undergoing carotid artery stenting. When bridging patients with the GP IIb/IIIA inhibitor tirofiban during intervention, the responder status did not influence post-interventional cerebral infarct patterns in this study.

## Supplemental Material

Supplemental Material - Drug response testing for elective carotid artery stenting: Prevalence of non-response to aspirin and clopidogrel and influence on post-interventional occurrence of cerebral ischemia Supplemental Material for Drug response testing for elective carotid artery stenting: Prevalence of non-response to aspirin and clopidogrel and influence on post-interventional occurrence of cerebral ischemia by Farzaneh Yousefi, Dimah Hasan, Frederic De Beukelaer, Hani Ridwan, Omid Nikoubashman, Martin Wiesmann, and Charlotte S. Weyland in The Neuroradiology Journal

## Appendix

### Abbreviations

ADC: apparent diffusion coefficient
CAS: carotid artery stenting
DAPT: dual antiplatelet therapy
DWI: diffusion weighted imaging
GP IIb/IIIA: glycoprotein IIb/IIIA
MRI: magnetic resonance imaging
PAD: peripheral arterial disease.
