# Supplementary material for: Drug response testing for elective carotid artery stenting: Prevalence of non-response to aspirin and clopidogrel and influence on post-interventional occurrence of cerebral ischemia
Source: Neuroradiol J. 2025 Aug 29:19714009251372360. Online ahead of print. doi: 10.1177/19714009251372360 (PMC12397092; doi:10.1177/19714009251372360)
Supplement: Supplemental Material - Drug response testing for elective carotid artery stenting: Prevalence of non-response to aspirin and clopidogrel and influence on post-interventional occurrence of cerebral ischemia [file sj-pdf-1-neu-10.1177_19714009251372360.pdf]

Supplementary materials:

Table 5 – frequency of protection method for the prevention of distal emboli during elective carotid artery stenting

Protection method	N (%)
Balloon guide catheter inflated during maneuvers	56 (82.4%)
Aspiration at guide catheter during balloon deflation or stent implantation	57 (83.8%)
filter wire placed distal to carotid artery stenosis	2 (2.9%)

Table 6 – influence of protection method for the prevention of distal emboli during elective carotid artery stenting on ischemic infarcts post intervention

	Balloon guide catheter asymp. Sig. (2-tailed)	aspiration asymp. Sig. (2-tailed)	filter wire asymp. Sig. (2-tailed)
Total infarctions, p-value	0.612	0.214	0.593
small infarctions, p-value	0.786	0.785	0.094
large infarctions, p-value	0.088	**0.007**	0.523
territorial infarctions, p-value	1.000	1.000	1.000
subcortical infarctions, p-value	0.699	0.169	0.983
cortical infarctions, p-value	0.435	0.155	0.591
deep grey matter, infarctions, p-value	0.116	0.136	0.555
Brainstem infarctions, p-value	1.000	1.000	1.000
